# Circulating alpha1-antitrypsin in the general population: Determinants and association with lung function

**DOI:** 10.1186/1465-9921-9-35

**Published:** 2008-04-25

**Authors:** Oliver Senn, Erich W Russi, Christian Schindler, Medea Imboden, Arnold von Eckardstein, Otto Brändli, Elisabeth Zemp, Ursula Ackermann-Liebrich, Wolfgang Berger, Thierry Rochat, Maurizio Luisetti, Nicole M Probst-Hensch, the SAPALDIA Team

**Affiliations:** 1 Molecular Epidemiology/Cancer Registry, Institutes of Social and Preventive Medicine & Clinical Pathology, University of Zurich, Zürich, Switzerland; 2 Pulmonary Division, University Hospital of Zurich, Zürich, Switzerland; 3 Institute of Social and Preventive Medicine, University of Basel, Basel, Switzerland; 4 Institute of Clinical Chemistry, University Hospital of Zurich, Zürich, Switzerland; 5 Zürcher Höhenklinik Wald, Wald, Switzerland; 6 Division of Medical Molecular Genetics and Gene Diagnostics, Institute of Medical Genetics, University of Zurich, Schwerzenbach, Switzerland; 7 Division of Pulmonary Medicine, University Hospital of Geneva, Geneva, Switzerland; 8 Clinica di Malattie dell'Apparato Respiratorio, Laboratorio di Biochimica e Genetica, IRCCS Policlinico San Matteo, Università di Pavia, Pavia, Italy

## Abstract

**Background:**

Severe alpha1-antitrypsin (AAT) deficiency associated with low AAT blood concentrations is an established genetic COPD risk factor. Less is known about the respiratory health impact of variation in AAT serum concentrations in the general population. We cross-sectionally investigated correlates of circulating AAT concentrations and its association with FEV1.

**Methods:**

In 5187 adults (2669 females) with high-sensitive c-reactive protein (CRP) levels ≤ 10 mg/l from the population-based Swiss SAPALDIA cohort, blood was collected at the time of follow-up examination for measuring serum AAT and CRP.

**Results:**

Female gender, hormone intake, systolic blood pressure, age in men and in postmenopausal women, as well as active and passive smoking were positively, whereas alcohol intake and BMI inversely correlated with serum AAT levels, independent of CRP adjustment. We observed an inverse association of AAT with FEV1 in the total study population (p < 0.001), that disappeared after adjustment for CRP (p = 0.28). In addition, the AAT and FEV1 association was modified by gender, menopausal status in women, and smoking.

**Conclusion:**

The results of this population-based study reflect a complex interrelationship between tobacco exposure, gender related factors, circulating AAT, systemic inflammatory status and lung function.

## Introduction

Alpha1-antitrypsin (AAT), a polymorphic inflammation-sensitive plasma protein with antiprotease activity, protects the lung tissue from destruction by neutrophil elastase. Severe and intermediate AAT deficiency is one of the most common inherited diseases on a global scale [1]. It increases the risk of chronic obstructive pulmonary diseases (COPD) in active smokers [2,3]. The two most common deficiency alleles are the S- and Z-allele, but at least 30 rare, additional alleles exist that are associated with reduced or absent plasma AAT levels [4].

International AAT deficiency registries have advanced the epidemiologic understanding of genetically determined AAT deficiency. Much less is known about the respiratory health impact of variation in AAT serum concentrations as observed in the general population. According to a study in healthy blood donors only 26% of the variance in circulating AAT was explained by known AAT gene variants [5]. In a population-based sample of school children pulmonary function was positively associated with C-reactive protein (CRP)-adjusted AAT levels, independent of PI phenotypes [6]. In contrast, an inverse association between inflammation-sensitive proteins, including non-CRP adjusted serum AAT, and pulmonary function has been described in population-based samples of adults [7,8]. These reported opposite directions of the lung function/AAT association may in part be due to differences in adjustment for CRP between the studies. They also agree with the dual role of AAT as both, an antiprotease and an inflammatory marker. Low-grade systemic inflammation is increasingly being recognized as a COPD risk factor [9].

In the population-based Swiss Cohort Study of Air Pollution and Lung Disease in Adults (SAPALDIA) we investigated the potential value of circulating AAT as a biomarker for susceptibility to respiratory disease in the general population. First, we identified environmental and lifestyle factors associated with AAT concentrations in the blood. Second, we investigated the cross-sectional association between circulating AAT concentrations and forced expiratory volume in one second (FEV1).

## Methods

### Study population

**Figure 1:**
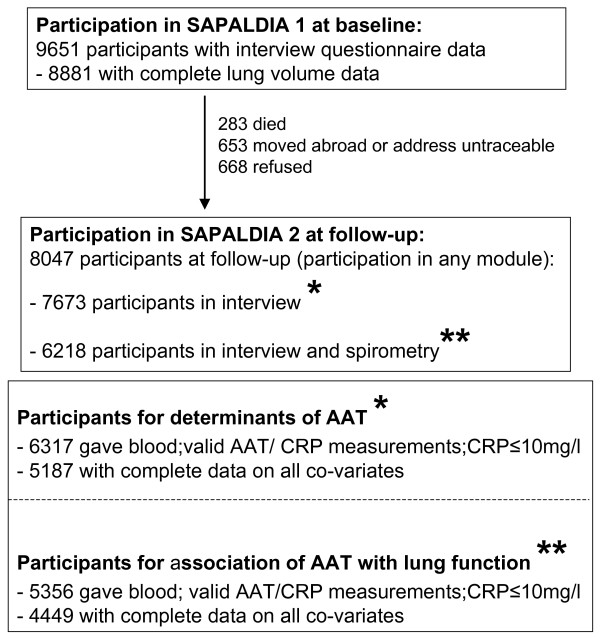
**Participation in SAPALDIA baseline and follow-up examination and in the current cross-sectional study.** The current, cross-sectional investigation of AAT is restricted to data collected in 2002/2003 when the blood bank was established. The analysis of factors correlated with serum AAT levels (Tables 2 and 7) is restricted to 5187 follow-up participants (*), whereas the association between serum AAT and FEV1 was further restricted to 4449 follow-up participants with valid lung function measurements (**) (Tables 4, 5 and 6).

The SAPALDIA cohort has been described [10,11]. At baseline in 1991 the SAPALDIA participants were aged 18–60 years. They are predominantly of European-Caucasian ethnicity and Swiss nationality. They were randomly selected from eight population registries representing the three major Swiss language regions and including both, urban and rural areas. Health examinations at baseline and after 11-years included an interview about respiratory health, occupational and lifestyle exposures as well as spirometry. Participation rate at baseline was 59.3%. The current, cross-sectional investigation of AAT is restricted to follow-up data collected in 2002/2003 when the blood bank was established (Figure 1 and Table 1 for more detail). Ethical approval for the study was given by the central ethics committee of the Swiss Academy of Medical Sciences and the Cantonal Ethics Committees for each of the eight examination areas.

**Table 1:** Characteristics at the baseline examination of participants in different parts of the study

	SAPALDIA baseline participants	SAPALDIA participants in study on AAT determinants	SAPALDIA participants in study on AAT and lung function
N	9651	5187	4449
Female, %	50.7	51.5	48.6
Age (mean, SD) [years]	41.7 (11.7)	40.9 (11.5)	41.1 (11.4)
BMI (mean, SD) [kg/m^2^]	24.0 (3.9)	23.6 (3.5)	23.7 (3.5)
Smoking Status, %			
- never	43.9	49.8	47.8
- former	22.6	20.6	22.0
- current	33.5	29.6	30.2
FEV1 (mean, SD) [ml/s]	3.53 (0.85)		3.59 (0.83)

### Measurements

#### Lung function

Lung function was assessed using spirometry (Sensormedics model 2200, Yorba Linda, USA) according to ATS criteria The present analysis focused on FEV1 expressed as % predicted, derived from SAPALDIA-specific prediction equation [12].

#### Tobacco exposure, alcohol intake and female hormone-related variables

Information about active and passive smoking was collected by an extended version of the European Community Respiratory Health Survey (ECRHS) questionnaire [13]. Smoking was categorized as current (smoking within month before interview), former, and never smoking at follow-up. Never and former smokers were classified as environmental tobacco smoke (ETS) exposed based on an affirmative answer to a question about regular exposure to ETS in the 12 months before the interview. Smoking intensity was calculated as total pack years smoked ([years of smoking * mean cigarettes/day]/20). Current smokers were *a priori *divided into two subgroups: <15 cigarettes/day vs. ≥ 15 cigarettes/day. Alcohol consumption was *a priori *classified as daily versus not daily drinking (≥1 vs. <1 alcoholic drink per day).

Women were categorized as pre- and postmenopausal. Women whose menses had become less regular within 12 month before the interview were defined as perimenopausal. Current exogenous hormonal intake was assessed by the questions "have you ever taken oral contraceptives (OC) or hormone replacement therapy (HRT)?", and "were you still taking OC or HRT during the last month before the interview?"

#### Physical examination

Mean values of 2 automatic measurements of systemic blood pressure (BP) (OMRON 705 CP, Tokyo, Japan) were used and pulse pressure (PP) – a measure of artery stiffness – was calculated as the difference between systolic and diastolic BP [14]. Weight and height were measured by electronic (TERRAILLON, Bradford, MA, USA), and telescopic scales (SECA, Hamburg, Germany), respectively for the assessment of body mass index (kg/m^2^; BMI).

#### Serum analysis

AAT (g/l) and high-sensitivity c-reactive protein (CRP) (mg/l) concentrations were determined by latex-enhanced immunoturbidimetric assays (Roche Diagnostics, Mannheim, Germany on a Roche COBAS INTEGRA analyzer, Rotkreuz, Switzerland) with interassay coefficients of variation below 5%. Lower detection rates for the AAT and CRP assays were 0.21 g/l, and 0.1 mg/l, respectively.

### Statistical analysis and sample size

All statistical analyses were restricted to subjects without signs of an acute infection (CRP ≤ 10 mg/l).

First, we identified the independent association of gender, age, BMI, smoking status, packyears, ETS exposure, and alcohol consumption with AAT concentrations among 5187 participants. Analysis of covariance with and without CRP adjustment was performed to estimate adjusted mean (SE) AAT serum concentrations (g/l).

Second, we investigated the association between circulating AAT and FEV1% predicted. Given the correlations of CRP with both, AAT (Table 2) and FEV1 (Table 3), we investigated the association with and without CPR-adjustment. The regression models included sex, study area, systolic blood pressure, body mass index (BMI), smoking status (never, former, current), alcohol consumption, ETS (never and former smokers), pack years smoked (current smokers), time since quitting (former smokers), cigarettes smoked/day (current smokers), menopausal status and hormone intake (women) as covariates. Analysis of covariance was performed to estimate adjusted mean FEV1% predicted values (standard errors) for AAT quintile classes. To evaluate dose-response relationships, predictor variables were categorized into suitable quantile classes, and regression models without the respective predictor variables were computed. A Cuzick's trend test [15] was then used to test whether regression residuals showed a monotonous association with increasing levels of the predictor in question. Effect modification was assessed by including interaction terms between AAT as a continuous variable and the potential effect modifier of interest in the models. Interactions with a p-value < 0.05 were considered statistically significant [16]. In the absence of information on *SERPINA1 *genotype variants and to avoid unrecognized confounding or effect modification by unrecognized severe and intermediate AAT deficiency genotypes, the main multiple linear regressions were restricted to participants exhibiting normal serum AAT concentrations (≥ 0.9 g/l; indicating the normal cut-off value for AAT serum concentrations according to the consensus reference data [CRM470/RPPHS] [17]). The main FEV1 analyses therefore included 4297 subjects (never smokers = 2028, former smokers = 1336, current smokers = 933) with complete data on lung function and covariates. Nevertheless and despite limited sample size, predicted means for the small category of subjects with serum AAT < 0.90 g/l (Q0: n = 152) are also presented in Tables 4, 5 and 6. STATA software, release 8.2 (StataCorp, TX, USA) was used for all statistical analyses.

**Table 2:** Adjusted* mean AAT serum levels by age, gender, anthropometrics, blood pressure, crp, and lifestyle variables (n = 5187)

		**Number of subjects**	**AAT (SE) (g/L), no CRP adjustment**	**AAT (SE) (g/L), CRP adjustment**
**Gender**	Men	2518	1.203 (0.004)	1.215 (0.004)
	Women	2669	1.305 (0.004)	1.293 (0.004)
			p < 0.001	p < 0.001
**Age**	Quartiles (years):			
**Men**	<43	630	1.194 (0.007)	1.195 (0.007)
	<53	629	1.200 (0.007)	1.205 (0.006)
	<61	630	1.208 (0.007)	1.208 (0.006)
	<73	629	1.239 (0.007)	1.233 (0.007)
			p trend < 0.001	p trend = 0.001
Premenopausal women^¶,†^	<38	202	1.314 (0.013)	1.312 (0.012)
	<43	201	1.284 (0.013)	1.284 (0.012)
	<50	202	1.278 (0.013)	1.281 (0.012)
	<55	201	1.227 (0.013)	1.227 (0.012)
			p trend < 0.001	p trend < 0.001
Postmenopausal women^¶,†^	<55	212	1.221 (0.014)	1.221 (0.013)
	<61	212	1.255 (0.013)	1.261 (0.012)
	<67	212	1.281 (0.013)	1.276 (0.013)
	<73	212	1.275 (0.013)	1.273 (0.013)
			p trend 0.05	p trend 0.07
**BMI**	Quartiles (kg/m^2^):			
	<22.79	1338	1.284 (0.005)	1.306 (0.005)
	<25.38	1349	1.250 (0.005)	1.257 (0.005)
	<28.36	1287	1.241 (0.005)	1.237 (0.005)
	≥28.36	1213	1.247 (0.005)	1.218 (0.005)
			p trend = 0.003	p trend < 0.001
**Systolic blood pressure**	Quartiles (mmHg):			
	<114	1342	1.240 (0.006)	1.244 (0.005)
	<126	1279	1.252 (0.005)	1.254 (0.005)
	<139	1304	1.260 (0.005)	1.259 (0.005)
	≥139	1262	1.272 (0.006)	1.266 (0.005)
			p trend < 0.001	p trend 0.002
**Tobacco exposure‡**	Never smokers/ETS-	2121	1.236 (0.005)	1.238 (0.004)
	Never smokers/ETS+	388	1.260 (0.010)	1.269 (0.009)
	Former smokers/ETS-	1188	1.238 (0.006)	1.240 (0.005)
	Former smokers/ETS+	312	1.249 (0.011)	1.249 (0.010)
	Current smokers, <15 cig/d)	515	1.284 (0.008)	1.285 (0.008)
	Current smokers, ≥15 cig/d)	662	1.328 (0.009)	1.314 (0.008)
			p trend < 0.0001	p trend < 0.0001
**Alcohol intake**	<1 drink per day	4069	1.261 (0.003)	1.262 (0.003)
	≥1 drink per day	1118	1.235 (0.006)	1.234 (0.007)
			p < 0.001	p < 0.001
**CRP**	Quartiles (mg/l):			
	<0.6	1506	1.182	-
	<1.1	1202	1.227	-
	<2.2	1232	1.268	-
	≥ 2.2	1247	1.360	-
			P trend < 0.00001	

*adjusted for age, gender, smoking status, alcohol intake, BMI, systolic blood pressure and study area ^¶^women taking oral contraceptives (n = 166), hormone replacement (n = 524), with perimenopausal status (n = 82), and unclear/missing hormonal status (n = 243) were excluded; ^†^p_(interaction) _pre-versus postmenopausal women <0.001; **‡ **n = 5186, due to 1 former smoker with missing ETS exposure; ETS = environmental tobacco exposure

**Table 3:** Association (z-value; p trend) of FEV1 %predicted with CRP quartile classes^§^

		z-value#	p_trend _(across crp quartiles)
**Non-smoking men**			
FEV1 (% predicted)*		-3.91	<0.000
FEV1 (% predicted)**		-4.53	<0.000
**Non-smoking women **¶			
FEV1 (% predicted)*		-2.32	0.02
FEV1 (% predicted)**		-2.15	0.03
**Smoking men**			
FEV1 (% predicted)*		-2.44	0.015
FEV1 (% predicted)**		-4.82	<0.000
**Smoking women **¶			
FEV1 (% predicted)*		-2.44	0.015
FEV1 (% predicted)**		-3.14	0.002

*controlled for BMI, systolic blood pressure, ETS (in non smokers), packyears (in smokers), alcohol intake, study area; ** crude associations; ¶additionally adjusted for menopausal status, and intake of female hormones; subjects with crp > 10 mg/l were excluded; §CRP quartiles (Q) (mg/l): Q1 < 0.6, Q2 < 1.1, Q3 < 2.2, Q4 > 2.2; # Regression models without CRP quartiles as predictor variable were computed first. A Cuzick's trend test was then used to test whether regression residuals showed a monotonous association with CRP quartiles. Under the null hypothesis of no trend, the z-value of Cuzick's trend test statistic approximately follows a standard normal distribution, enabling the computation of approximate p-values; a negative sign indicates an inverse association between CRP and FEV1%predicted [15].

**Table 4:** Mean (SE) FEV1 %predicted in relation to AAT quintile classes †

	Quintile classes of AAT (g/l)						
	*Q0 (<0.9)*	Q1 (≥0.9–<1.13)	Q2 (≥1.13, <1.22)	Q3 (≥1.22, <1.31)	Q4 (≥1.31, <1.41)	Q5 (≥1.41)	p_trend _from model not including Q0

**All (n) **¶	*152*	952	832	932	764	817	
FEV1 (% predicted) CRP adj.	*99.8 (1.2)*	98.0 (0.5)	98.3 (0.5)	98.1 (0.5)	97.6 (0.5)	96.6 (0.5)	0.28
FEV1 (% predicted), no CRP adj. *	*100.4 (1.2)*	98.7 (0.5)	98.7 (0.5)	98.2 (0.5)	97.4 (0.5)	95.5 (0.5)	**<0.001**
							
**Men (n)‡**	*79*	627	511	460	351	256	
FEV1 (% predicted) CRP adj.	*99.0 (1.6)*	96.7 (0.6)	97.3 (0.6)	97.2 (0.6)	95.8 (0.8)	93.8 (1.0)	0.08
FEV1 (% predicted), no CRP adj. *	*99.3 (1.6)*	97.3 (0.6)	97.6 (0.6)	97.1 (0.7)	95.4 (0.8)	92.6 (0.9)	**<0.001**
							
**Women (n) **¶, ‡	*73*	325	321	472	413	561	
FEV1 (% predicted) CRP adj.	*100.6 (1.6)*	99.4 (0.8)	99.2(0.8)	99.3(0.7)	99.2 (0.7)	98.8 (0.6)	0.76
FEV1 (% predicted), no CRP adj. *	*101.4 (1.7)*	99.9 (0.8)	99.7(0.8)	99.5(0.7)	99.3 (0.7)	97.8 (0.6)	0.19
							
**Never/Former Smokers (n) **¶, ‡	*130*	811	721	754	541	537	
FEV1 (% predicted), CRP adj.	*100.4 (1.3)*	99.1 (0.5)	99.0 (0.5)	99.1 (0.5)	98.4 (0.6)	96.9 (0.7)	0.10
FEV1 (% predicted), no CRP adj.*	*100.9 (1.3)*	99.6 (0.5)	99.3 (0.5)	99.1 (0.5)	98.2 (0.6)	95.9 (0.7)	**<0.001**
							
**Current Smokers (n) **¶, ‡	*22*	141	111	178	223	280	
FEV1 (% predicted), CRP adj.	*96.9 (2.9)*	93.6 (1.2)	95.0 (1.3)	93.8 (1.0)	94.5 (0.9)	95.6 (0.9)	0.16
FEV1 (% predicted), no CRP adj.*	*97.2 (2.9)*	94.3 (1.0)	95.7 (1.3)	94.3 (1.0)	94.7 (0.9)	94.5 (0.9)	0.91
							
**Currents Smokers ≥15 cigs/d (n) ¶,‡**	*11*	60	48	103	122	182	
FEV1 (% predicted), CRP adj.	*96.5 (4.3)*	88.8 (1.9)	93.6 (2.1)	91.2 (1.4)	93.6 (1.3)	95.3 (1.1)	**0.01**
FEV1 (% predicted), no CRP adj.*	*96.7 (4.3)*	89.7 (1.9)	94.4 (1.1)	91.2 (1.4)	93.9 (1.3)	94.4 (1.1)	0.06
							
**Currents Smokers <15 cigs/d (n) ¶,‡**	*11*	81	63	75	101	98	
FEV1 (% predicted), CRP adj.	*99.6 (3.9)*	98.3 (1.5)	96.9 (1.7)	96.6 (1.5)	95.8 (1.3)	94.9 (1.5)	0.21
FEV1 (% predicted), no CRP adj.*	*99.9 (3.9)*	98.9 (1.5)	97.6 (1.4)	97.2 (1.5)	95.9 (1.3)	93.5 (1.4)	**0.02**

† adjusted for gender, smoking status (never, former, current), years since quitting in former smokers, cigarettes/day in current smokers, passive smoking in never smokers, BMI, systolic blood pressure, alcohol consumption, study area, menopausal status in women, intake of female hormones in women, CRP, CRP^2 ^(models with CRP adjustment); * adjusted for all covariates but CRP; ¶women in perimenopausal status (n = 35) and missing/unclear status and hormonal intake (n = 120) were excluded from the analysis; ‡ P-values for interactions: In All: AAT*gender: p = 0.02(CRP adj); p = 0.007(no CRP adj.); In women: AAT*menopausal status: 0.01 (CRP adj.); 0.008 no CRP adj.); AAT*intake female hormones: p > 0.80, irrespective of menopausal status; AAT*smoking (heavy current smoking vs. all others): all men: p = 0.02 (CRP adj.); p = 0.03 (no CRP adj.); all women: p = 0.11 (CRP adj.); p = 0.11 (no CRP adj.); postmenopausal women: p = 0.08 (CRP adj.); p = 0.07 (no CRP adj.); premenopausal women: p = 0.59 (CRP adj.); p = 0.58 (no CRP adj.)

**Table 5:** Mean (SE) FEV1 %predicted in relation to AAT quintile classes in non-current smokers†

	Quintile classes of AAT (g/l)						
	*Q0 (<0.9)*	Q1 (≥0.9–<1.13)	Q2 (≥1.13, <1.22)	Q3 (≥1.22, <1.31)	Q4 (≥1.31, <1.41)	Q5 (≥1.41)	p_trend _from model not including Q0

**Men (n)**	*66*	522	439	351	226	142	
FEV1 (% predicted), CRP adj.	*99.5 (1.8)*	97.6 (0.6)	97.9 (0.7)	98.2 (0.8)	97.0 (1.0)	92.9 (1.3)	**0.05**
FEV1 (% predicted), no CRP adj.*	*99.8 (1.8)*	98.1 (0.6)	98.1 (0.6)	98.0 (0.8)	96.5 (0.9)	91.9 (1.2)	**<0.001**
**Women (n) **¶	*64*	289	282	403	315	395	
FEV1 (% predicted) CRP adj.	*101.0 (1.8)*	100.4 (0.9)	99.8 (0.9)	100.3 (0.7)	99.9 (0.8)	99.3 (0.8)	0.85
FEV1 (% predicted), no CRP adj. *	*101.8 (1.8)*	100.9 (0.9)	100.3 (0.9)	100.4 (0.7)	100.0 (0.8)	98.4 (0.7)	0.13
**Premenopausal women (n) **¶	*27*	100	109	151	131	187	
FEV1 (% predicted), CRP adj.	*98.7 (2.4)*	98.3 (1.3)	97.2 (1.2)	99.6 (1.0)	101.5 (1.1)	99.7 (1.0)	**0.02**
FEV1 (% predicted), no CRP adj.*	*99.1 (2.4)*	98.8 (1.3)	97.1 (1.0)	99.6 (1.0)	101.7 (1.1)	99.1 (1.0)	0.11
**Postmenopausal women (n) **¶	*37*	189	173	252	184	208	
FEV1 (% predicted), CRP adj.	*102.5 (2.5)*	101.5 (1.1)	101.5 (1.2)	100.8 (1.0)	98.8 (1.1)	98.7 (1.1)	**0.04**
FEV1 (% predicted), no CRP adj.*	*103.4 (2.5)*	102.1 (1.1)	101.7 (1.1)	101.0 (1.0)	98.7 (1.1)	97.7 (1.1)	**<0.001**

† adjusted for BMI, passive smoking, years since quitting, systolic blood pressure, alcohol consumption, study area, CRP, CRP^2 ^(models with CRP); * adjusted for all covariates but CRP; ¶additionally adjusted for menopausal status, intake of female hormones; women in perimenopausal status (n = 35) and missing/unclear status and hormonal intake (n = 120) were excluded;

**Table 6:** Mean (SE) FEV1 %predicted in relation to AAT quintile classes in current smokers †

	Quintile classes of AAT (g/l)						
	*Q0 (≤ 0.9)*	Q1 (≥0.9–<1.13)	Q2 (≥1.13, <1.22)	Q3 (≥1.22, <1.31)	Q4 (≥1.31, <1.41)	Q5 (≥1.41)	p_trend _from model not including Q0

**Men (n)**	*13*	105	72	109	125	114	
FEV1 (% predicted), CRP adj.†	*95.1 (3.8)*	92.8 1.4)	93.8 (1.7)	93.4 (1.3)	93.2 (1.2)	95.4 (1.4)	0.30
FEV1 (% predicted), no CRP adj.*	*95.6 (3.8)*	93.3 (1.4)	94.1 (1.7)	93.8 (1.3)	93.3 (1.2)	94.3 (1.3)	0.71
							
*Men ≥15 cigs/day (n)*	*6*	47	33	72	72	87	
FEV1 (% predicted), CRP adj.†	*95.3 (5.7)*	89.3 (2.1)	93.3 (2.5)	91.2 (1.7)	93.7 (1.6)	95.5 (1.6)	**0.04**
FEV1 (% predicted), no CRP adj.*	*89.7 (2.1)*	89.7 (2.1)	93.3 (2.5)	91.4 (1.7)	93.8 (1.7)	94.9 (1.6)	**0.07**
							
*Men <15 cigs/day (n)*	*7*	58	39	37	53	27	
FEV1 (% predicted), CRP adj.†	*96.5 (5.0)*	96.0 (1.8)	94.3 (2.2)	96.6 (2.2)	92.99 (1.9)	94.1 (2.8)	0.38
FEV1 (% predicted), no CRP adj.*	*97.3 (2.2)*	96.7 (1.7)	95.2 (2.2)	97.0 (2.2)	92.5 (1.9)	91.1 (2.6)	**0.05**
							
**Women (n) **¶	*9*	36	39	69	98	166	
FEV1 (% predicted), CRP adj.†	*98.6 (4.5)*	94.6 (2.3)	96.8 (2.2)	94.1 (1.6)	95.9 (1.3)	96.7 (1.1)	0.24
FEV1 (% predicted), no CRP adj.*	*98.6 (4.5)*	95.5 (2.3)	92.2 (2.2)	94.9 (1.6)	96.4 (1.4)	95.5 (1.1)	0.99
*Premenopausal (n)*	*4*	21	21	32	40	81	
FEV1 (% predicted), CRP adj.†	*98.8 (6.2)*	96.1 (2.7)	95.4 (2.7)	97.0 (2.2)	100.2 (2.0)	94.6 (1.4)	0.71
FEV1 (% predicted), no CRP adj.*	*98.8 (6.2)*	96.7 (2.7)	95.3 (2.2)	99.0 (1.9)	101.0 (1.9)	93.6 (1.4)	0.64
*Postmenopausal (n)*	*5*	15	18	37	58	85	
FEV1 (% predicted), CRP adj.†	*98.3 (6.4)*	91.7 (3.7)	97.7 (3.4)	92.4 (2.3)	92.3 (1.8)	99.2 (1.6)	0.15
FEV1 (% predicted), no CRP adj.*	*97.1 (6.6)*	93.7 (3.7)	99.6 (3.4)	93.5 (2.4)	92.7 (1.9)	97.7 (1.6)	0.69
							
***Postmenopausal, ≥ 15 cigs/day (n))***¶	*4*	8	7	19	31	53	
FEV1 (% predicted), CRP adj.†	*94.6 (8.3)*	84.7 (5.5)	90.0 (6.0)	87.9 (3.6)	90.0 (2.7)	96.3 (2.2)	**0.05**
FEV1 (% predicted), no CRP adj.*	*93.9 (8.5)*	87.4 (2.2)	92.7 (6.2)	88.1 (3.7)	90.4 (2.9)	95.1 (2.2)	0.22
							
*Postmenopausal, <15 cigs/day (n)*¶	*1*	7	11	18	27	32	
FEV1 (% predicted), CRP adj.†	*105.4 (12.3)*	100.2 (4.6)	103.6 (3.8)	98.4 (3.0)	96.2 (2.3)	102.3 (2.4)	0.85
FEV1 (% predicted), no CRP adj.*	*108.8 (12.6)*	100.2 (4.7)	103.5 (3.8)	99.1 (3.0)	96.7 (2.3)	101.3 (2.2)	0.93

## Results

At baseline 9651 subjects were recruited into the SAPALDIA cohort, of whom 8881 underwent lung function testing (Figure 1). Of the 7673 participants in the interview at follow-up (Fig.1, see *), 5187 were included in the analysis of factors associated with serum AAT (Table 2 and 7). Equivalently, of the 6218 participants in both, interview and spirometry at follow-up (Fig. 1, see **), 4449 were included in the analysis of the serum AAT/FEV1% predicted association (Tables 4, 5 and 6). A comparison of baseline characteristics between all SAPALDIA participants (n = 9651) and subjects included in the two current sub-studies (AAT determinants (n = 5187); AAT/FEV1% predicted (n = 4449) is listed in Table 1. As previously described in detail, never smokers were overrepresented among participants at follow-up and thus in the two cross-sectional sub-studies [11].

**Table 7:** Adjusted* mean AAT serum levels in women by menopausal status and hormone intake

	**Predictor**	**Number of subjects**	**AAT (SE) (g/L) no CRP adjustment**	**AAT (SE) (g/L) CRP adjustment**
Menopausal status in women without hormonal treatment^¶,†^:	Premenopausal	806	1.277 (0.007)	1.282 (0.007)
	Postmenopausal	848	1.256 (0.007)	1.252 (0.006)
			p = 0.038	p = 0.003
Oral contraceptives in premenopausal women:	no OC use	640	1.278 (0.007)	1.288 (0.007)
	use of OC	166	1.520 (0.016)	1.470 (0.016)
			p < 0.001	p < 0.001
Hormone replacement in postmenopausal women:	no use of HRT	324	1.207 (0.015)	1.216 (0.014)
	use of HRT	524	1.272 (0.015)	1.268 (0.014)
			p < 0.001	p < 0.001

* adjusted for BMI, age, active and passive smoking, alcohol consumption, systolic blood pressure and study area ^¶^perimenopausal women (n = 82) and women with unclear/missing information on hormonal use (n = 243) were excluded; ^†^not adjusted for age due to co-linearity between age and menopausal status; OC = oral contraception; HRT = hormone replacement therapy;

### AAT serum levels: association with age, gender, anthropometrics, blood pressure, lifestyle, and hormonal variables

At the follow-up examination, mean (SD) age in men (n = 2518) and women (n = 2669) included in the investigation of AAT determinants was 51 [12] and 52 [11] years, respectively, and mean BMI 26.45 (3.72) and 24.96 (4.55) kg/m^2^, respectively. In both gender groups combined, 23% reported current and 29% reported former smoking at follow up. Mean (SD) pack years smoked in current and former smokers was 30 [23] and 21 [23] pack years, respectively. Two thousand five hundred and nine subjects had never smoked actively (59% females), of whom 15% were exposed to environmental tobacco smoke (ETS) within 12 months before the follow-up interview. Among these, 137 (35%) were exposed to ETS more than 3 hours per day. Among the premenopausal women, 166 (21%) reported oral intake of contraceptives, and hormone replacement therapy was reported by 524 (61%) of the postmenopausal women. Serum AAT concentrations ranged from 0.38 to 2.24 g/l with a mean (SD) of 1.26 (0.20) g/l. One hundred and seventy eight subjects (3.4%) had a serum AAT level below 0.9 g/l, the recommended test limit for further clinical and laboratory investigation of genetic AAT deficiency [17].

The associations of age, gender, anthropometric parameters, blood pressure, lifestyle and hormonal variables with serum AAT are shown in Tables 2 and 7. Women had higher circulating AAT concentrations (p < 0.001) (Table 2).

The AAT/age association differed by gender and menopausal status in women (p interaction <0.001 for both). In men and postmenopausal women age was positively associated with AAT whereas an inverse association was present in premenopausal women. None of the remaining AAT predictors was modified by gender. Systolic blood pressure and pulse pressure (data not shown) were positively associated with serum AAT (p trend = 0.002 and <0.001, respectively), BMI and regular alcohol consumption inversely. Serum AAT increased with tobacco smoke exposure in a dose-dependent fashion. ETS-exposed never smokers had higher AAT serum levels than non-exposed never smokers. Serum AAT was highest in active smokers consuming at least 15 cigarettes per day (p linear trend < 0.001).

In women, we assessed the association of menopausal status and intake of female hormones (oral contraceptives or hormone replacement therapy) with serum AAT (Table 7).

Among women without intake of female hormones, serum AAT was higher in premenopausal women (p = 0.003). Use of oral contraceptives (premenopausal) and of hormone replacement therapy (postmenopausal) was associated with elevated AAT concentrations in the blood (p < 0.001 for both).

The associations of serum AAT with the factors listed in Tables 2 and 7 were insensitive to CRP adjustment. CRP itself was positively associated with AAT (Table 2). Results in Tables 2 and 7 were insensitive to the exclusion of subjects with AAT levels below 0.9 g/l or below 1.04 g/l (0.9 g/l: normal cut-off for serum AAT [17]; 1.04 g/l: lower 10^th ^percentile of the AAT distribution in this study).

### AAT serum levels: association with lung function

In the total study population, AAT was inversely associated with FEV1, but only in the absence of CRP adjustment (Table 4).

The inverse association was restricted to men (not CRP adjusted p interaction _AAT*gender _= 0.007). The AAT/FEV1 association was also modified by smoking. An inverse association before CRP adjustment was observed in never/former smokers (p < 0.001) and in current smokers consuming 15 cigarettes or less per day. In contrast, CRP adjusted AAT and FEV1 were positively correlated in heavy current smokers (p = 0.01) (not CRP adjusted p interaction _AAT*(current smokers >= 15 cigs/day vs. all others)_: = 0.14 in all subjects; = 0.03 in men; = 0.11 in women).

Results on the AAT/FEV1 association stratified by both, gender and smoking, are presented in Tables 5 and 6. As we observed a statistically significant interaction between menopausal status and AAT in all women, irrespective of smoking status and CRP adjustment (not CRP adjusted p interaction _AAT*menopausalstatus _= 0.008), analyses in women were further stratified by menopausal status.

Among participants not currently smoking, FEV1 was inversely related to AAT in both, men and postmenopausal women, irrespective of CRP adjustment (Table 5).

In contrast, premenopausal non-smoking women exhibited a positive correlation between FEV1 and CRP-adjusted serum AAT (p = 0.02).

No inverse associations between FEV1 and AAT were observed in current smokers upon stratification by gender (Table 6).

Upon stratification by smoking intensity (< vs. ≥ 15 cigs/day) the association between AAT and lung function became positive in the group of heavy smokers after adjustment for CRP in both, men and postmenopausal women (men: p = 0.04; women: p = 0.05). Unfortunately, we had insufficient power to stratify analysis in premenopausal women by smoking intensity.

Analyses of the AAT/FEV1 associations were also repeated by increasing the exclusion cutoff to AAT levels below 1.04 g/l (lower 10^th ^percentile of the AAT distribution in this study). The results presented in Tables 4, 5 and 6 were insensitive to this additional exclusion. It is notable that subjects with AAT < 0.9 g/l generally exhibited good lung function (Tables 4, 5 and 6), although data in this category was sparse.

## Discussion

The main and general message of the present paper is that circulating AAT was inversely correlated with FEV1 in this general adult population, but only in the absence of CRP adjustment. The strengths of this study include its population-based design, and the detailed characterization of the participants which allowed us to investigate both, factors associated with circulating AAT as well as the association of the latter with lung function. Based on CRP measurements we were able to exclude subjects with laboratory evidence of inflammation at the time of the blood draw, and to control for low-grade systemic inflammation as potential confounder. These study advantages allowed us to demonstrate the complexity of the interrelationship between tobacco exposure, gender, circulating AAT and CRP as well as lung function.

Positive associations between active smoking and AAT levels have been reported before [18,19]. We additionally considered ETS and smoking intensity and demonstrated a positive dose-response relationship between tobacco exposure and serum AAT, that was not confounded by CRP. But this positive association between tobacco exposure and circulating AAT did not consistently translate into an inverse association between AAT and lung function in smokers. In fact we found a positive AAT/FEV1 association in men and postmenopausal women who smoked heavily, in line with the interaction of smoking and inherited AAT deficiency on COPD risk and lung function [20]. Von Ehrenstein et al [6] found adverse effects of ETS on lung function to be strongest in children with low, CRP-adjusted AAT levels. The differences in FEV1 that we observed were small from an individual perspective; yet even small differences in FEV1 at normal levels are predictive of overall or cause-specific mortality and may ultimately shift a substantial percentage of the general population into the group of subjects with clinically relevant lung function deficits [21].

Our results suggest that female hormones influence circulating AAT levels and modify the AAT/lung function association. Gender differences in both, circulating AAT [20] and respiratory health [22] are well established and may reflect biological differences in the interrelations between smoking, AAT and lung function. Gender differences in AAT levels were even present in the situation of severe AAT deficiency although the results were not adjusted for hormone intake [23]. While previous reports on the association between use of oral contraceptives and AAT in the blood exist [24,25], our study extends this association to postmenopausal hormone intake. Our findings are compatible with an effect of estrogens or progesterone on hepatic AAT metabolism, which may be mediated by an inhibition of cytochrome P-450 activities [26].

The observed modification of the FEV1/AAT association by smoking and hormonal factors as well as by CRP-adjustment may reflect the dual role of AAT as a respiratory disease biomarker. The net impact of AAT on lung function seems to be the result of context-dependent (i.e. smoking, gender) and contrasting protective and inflammatory effects in the respiratory tract. On the one hand, elevated serum AAT can reflect a beneficial shift of the protease-antiprotease balance, the centre piece of the pathophysiological pathway mediating the effect of severe AAT deficiency on COPD. On the other hand elevated serum AAT can also reflect low-grade inflammatory processes in the lung [27], a hypothesized COPD risk factor [9]. Consistent with these findings, we could detect a positive relationship between AAT and CRP concentrations. Previous studies observed inverse association between inflammation-sensitive proteins, including serum AAT, and pulmonary function in population-based samples of adults [7,8]. Subjects with CRP > 10 mg/l were not excluded, and smoking was not investigated as a potential effect modifier. Significantly higher AAT levels were even reported for AAT deficient (PIZZ) patients with COPD compared to PIZZ individuals without COPD thus further supporting the hypothesis that AAT levels may also represent an ongoing inflammatory process [28].

The observed inverse association observed between AAT and BMI might also be reflecting different roles of AAT. In general, positive association between CRP and BMI have been observed among obese persons, an effect that was attributed to low-grade systemic inflammation [29]. However, the acute phase response is a complex signalling pathway and different acute phase proteins are distinctly regulated and expressed [30]. Engström et al [31] found a number of inflammation-sensitive proteins to be positively related to BMI, yet AAT levels were highest in healthy men in the lowest BMI quartile.

The cross-sectional nature of the study prohibits assessment of cause and effect as well as differentiation between etiological and prognostic effects of variation in AAT concentrations. This aspect may be of relevance to considerations about the pathophysiological mechanism underlying the observed associations. Dahl et al[20] found the AAT MZ genotype to be associated with lung function among individuals with clinically established COPD, but not among subjects without COPD in the general population. A possible criticism to our paper is the lack of information about potential AAT deficiency pheno- or genotypes in the study population. But we specifically investigated the potential value of variation at the serum AAT protein levels as a biomarker for lung function impairment in the general population. Since the observed associations were insensitive to the AAT cut-off level rare, unrecognized AAT deficiency alleles are an unlikely source of bias in this study. Another concern of the current cross-sectional study, which is embedded into the SAPALDIA cohort, is the potential for selection bias due to non-participation. Never smokers were more likely to participate at follow-up [11]. If serum AAT was associated with the likelihood of participation at baseline or follow-up this could have distorted results on associations between AAT and other factors. Although unlikely, we cannot definitively rule out such bias in the absence of AAT measurements in non-participants.

## Conclusion

Our cross-sectional results demonstrated that a complex interrelationship among tobacco exposure, gender, circulating AAT, lung function, and systemic inflammatory status exists. If the observed interactions between these variables are confirmed in larger and longitudinal studies, the potential utility of circulating AAT as a biomarker for susceptibility to respiratory disease seems limited. The reported interactions are also relevant to studying the impact of genetic variation in AAT on lung health.

## Competing interests

The authors declare that they have no competing interests.

## Authors' contributions

OS conducted the analysis and drafted the article. EWR, MI, AvE, CS, OB, EZ, UA, WB, TR, and NMPH contributed to the design of the study, the acquisition of data and interpretation of data. CS, EWR, TR, ML and NMPH also advised on the conduct of the analysis. All authors made important intellectual contributions during the drafting process and have given approval for the final version.

## SAPALDIA Team

### Study directorate

T Rochat (p), U Ackermann-Liebrich (e), JM Gaspoz (c), P Leuenberger (p), LJS Liu (exp), NM Probst Hensch (e/g), C Schindler (s).

### Scientific team

JC Barthélémy (c), W Berger (g), R Bettschart (p), A Bircher (a), G Bolognini (p), O Brändli (p), M Brutsche (p), L Burdet (p), M Frey (p), MW Gerbase (p), D Gold (e/c/p), W Karrer (p), R Keller (p), B Knöpfli (p), N Künzli (e/exp), U Neu (exp), L Nicod (p), M Pons (p), E Russi (p), P Schmid-Grendelmeyer (a), J Schwartz (e), P Straehl (exp), JM Tschopp (p), A von Eckardstein (cc), JP Zellweger (p), E Zemp Stutz (e).

### Scientific team at coordinating centers

PO Bridevaux (p), I Curjuric (e), SH Downs (e/s), D Felber Dietrich (c), A Gemperli (s), D Keidel (s), M Imboden (g), P Staedele-Kessler (s), GA Thun (g)

(a) allergology, (c) cardiology, (cc) clinical chemistry, (e) epidemiology, (exp) exposure, (g) genetic and molecular biology, (m) meteorology, (p) pneumology, (s) statistics
